# Good laboratory practices guarantee biosafety in the Sierra Leone-China friendship biosafety laboratory

**DOI:** 10.1186/s40249-016-0154-5

**Published:** 2016-06-23

**Authors:** Qin Wang, Wei-Min Zhou, Yong Zhang, Huan-Yu Wang, Hai-Jun Du, Kai Nie, Jing-Dong Song, Kang Xiao, Wen-Wen Lei, Jian-Qiang Guo, He-Jiang Wei, Kun Cai, Yan-Hai Wang, Jiang Wu, Gerard Kamara, Idrissa Kamara, Qiang Wei, Mi-Fang Liang, Gui-Zhen Wu, Xiao-Ping Dong

**Affiliations:** Chinese Center for Disease Control and Prevention, National Institute for Viral Diseases Control and Prevention, Beijing, China; Sierra Leone-China Friendship Biological Safety Laboratory, Freetown, Sierra-Leone; Key Laboratory for Medical Virology, National Health and Family Planning Commission, Beijing, China; State Key Laboratory for Infectious Disease Prevention and Control, Chinese Center for Disease Control and Prevention, Beijing, China; Chinese Center for Disease Control and Prevention, Beijing, China; Collaborative Innovation Center Diagnosis and Treatment of Infectious Diseases, Zhejiang University, Hangzhou, China; Chinese Academy of Sciences Key Laboratory of Pathogenic Microbiology and Immunology, Institute of Microbiology, Chinese Academy of Sciences, Beijing, China

**Keywords:** Ebola virus, Ebola virus disease, Biosafety, SLE-CHN Biosafety Lab, BSL-3 laboratory

## Abstract

**Background:**

The outbreak of Ebola virus disease (EVD) in West Africa between 2014 and 2015 was the largest EDV epidemic since the identification of Ebola virus (EBOV) in 1976, and the countries most strongly affected were Sierra Leone, Guinea, and Liberia.

**Findings:**

The Sierra Leone-China Friendship Biological Safety Laboratory (SLE-CHN Biosafety Lab), a fixed Biosafety Level 3 laboratory in the capital city of Sierra Leone, was established by the Chinese government and has been active in EBOV detection since 11 March 2015. Complete management and program documents were created for the SLE-CHN Biosafety Lab, and it was divided into four zones (the green, yellow, brown, and red zones) based on the risk assessment. Different types of safe and appropriate personnel protection equipment (PPE) are used in different zones of the laboratory, and it fully meets the Biosafety Level 3 laboratory standards of the World Health Organization.

**Conclusion:**

Good preparedness, comprehensive risk assessment and operation documents, appropriate PPE, effective monitoring and intensive training, together with well-designed and reasonable laboratory sectioning are essential for guaranteeing biosafety.

**Electronic supplementary material:**

The online version of this article (doi:10.1186/s40249-016-0154-5) contains supplementary material, which is available to authorized users.

## Multilingual abstracts

Please see Additional file 1 for translations of the abstract into the six official working languages of the United Nations.

## Background

The Ebola virus disease (EVD) outbreak that occurred in West Africa between 2014 and 2015 was the largest EDV epidemic since the identification of Ebola virus (EBOV) in 1976 [1–3]. The countries most strongly affected were Guinea, Liberia, and Sierra Leone, accounting for 99 % of EVD cases worldwide [4–7]. Shortly after EDV emerged in West Africa in 2014, the Chinese government offered to provide large-scale medical aid to the three most strongly affected countries [8, 9]. The aid included establishment of a fixed Biosafety Level 3 (BSL-3) laboratory in Freetown, the capital city of Sierra Leone. The project began on 20 November 2014 and construction was completed on 31 January 2015. The first round of fixed laboratory team from National Institute for Viral Disease Control and Prevention (NIVDC), Chinese Center for Disease Control and Prevention (China CDC) arrived in Sierra Leone on 8 February 2015. This laboratory, called the Sierra Leone-China Friendship Biological Safety Laboratory (SLE-CHN Biosafety Lab) has been performing EBOV detection since 11 March 2015.

## Layout of the SLE-CHN biosafety lab

**Fig. 1:**
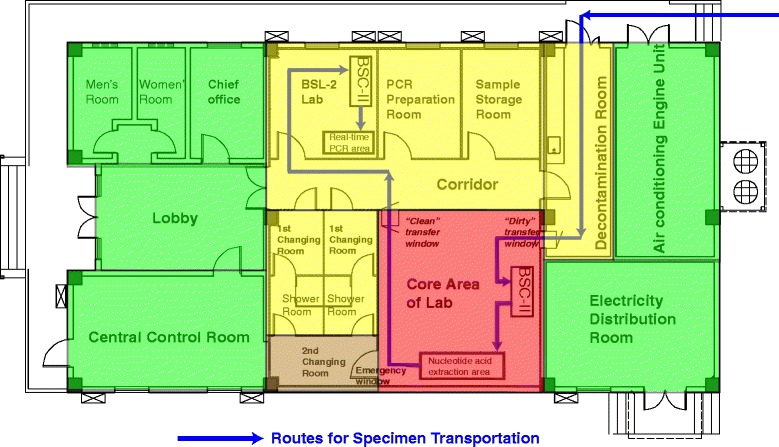
Layout of Sierra Leone-China Friendship Biological Safety Laboratory. The SLE-CHN Biosafety Lab mainly consists of a BSL-3 laboratory with negative pressure, a BSL-2 laboratory, a PCR preparation room, a storage room and two sets of dressing rooms (including two 1^st^ changing rooms, two shower rooms and one 2^nd^ changing room). The BSL-3 lab is connected to other labs through two transfer windows (a “clean” transfer window and a “dirty” transfer window). In addition, it contains a central control room and offices on the left side, and a decontamination room, an electricity distribution room and an air conditioning engine unit on the right side. The lab was divided into four zones, including a green zone, a yellow zone, a brown zone, and a red zone, which are indicated in the corresponding colours. Blue lines show the routes for specimen transportation through the lab

As shown in Fig. 1, the SLE-CHN Biosafety Lab mainly consists of a BSL-3 laboratory with negative pressure, a BSL-2 laboratory, a PCR preparation room, a storage room and two sets of dressing rooms (including two 1^st^ changing rooms, two shower rooms and one 2^nd^ changing room). The BSL-3 laboratory is connected to the other labs through two transfer windows (a “clean” transfer window and a “dirty” transfer window). In addition, the lab contains a central control room and offices on the left side, and a decontamination room, an electricity distribution room, and an air conditioning engine unit on the right side. The pressure in the BSL-3 laboratory is recorded by checking the pressure gauge in the lab every day, and it was also real-time monitored in the central control room. The air flow in BSL-2 laboratory is also monitored in the central control room. The generator, water supplying unit and incineration units are located outside the building. The SLE–CHN Biosafety Lab fully meets the biosafety level 3 laboratory standards of the World Health Organization (Fig. 1) [10].

## Document management for the SLE-CHN Biosafety Lab

Prior to arrival in Sierra Leone, a set of laboratory management and program documents were prepared based on the BSL-3 documents of the NIVDC, China CDC. Those documents were revised after we arrived at the SLE–CHN Biosafety Lab, according to the acute situation. The documents are comprised of four layers, including the management manual (30 files, describe the quality management system documentation), program documents (50 files, describe the function of quality requirement allocation), standard operation procedures (SOP) (20 files, describe the operation procedures, inspection standard, etc.), and working manual (29 files, describe the records of operation results). All documents were reviewed and issued by the Biosafety Committee of NIVDC.

## Functional sectorisation and operating procedure optimisation

Risk assessments help us ensure the hazards will not happen, we may not be able to eliminate all the hazards, however, the probability of incidence occurring can be minimized through risk assessment. Based on the risk assessment for EBOV, the SLE-CHN Biosafety Lab was sectioned into a green zone, which had no risk of contamination [including the central control room, offices, lobby (where the laboratory staffs getting into the BSL-3 lab), electricity distribution room, and air conditioning engine unit]; a yellow zone, which had the potential for contamination with EBOV nucleic acids (including the BSL-2 lab, the PCR preparation room, the storage room, the corridor, the 1^st^ changing room, the shower room, and the decontamination room); a brown zone, which has the potential for contamination with EBOV nucleic acids and an extremely low risk of contamination with live EBOV (2^nd^ changing room); and a red zone, which has the potential for contamination with live EBOV (BSL-3 lab) (Fig. 1).

The operating procedures and routes for EBOV detection were carefully draw up. Briefly, samples from patients suspected to have EVD were transferred into the BSL-3 lab in a specimen container via the “dirty” transfer window, after decontamination of the outside of the container with 0.5 % chlorine-containing disinfectant. The processes of package unwrapping, sample registration and photographing, sample aliquoting, EBOV inactivation [11], and a blood assay for malaria (colloidal gold method) were performed in the biosafety cabinet in the BSL-3 laboratory. The inactivated samples were transferred to the RNA extraction bench for either manual or automatic RNA extraction. The extracted RNA containing Eppendorf tubes that in a corrosion-resistant box had been soaked in disinfectant for at least 10 min for external disinfection, were then transferred to the BSL-2 lab via the “clean” transfer window for EBOV detection using a commercial real-time RT-PCR kit for EBOV (Zaire strain) (Registration No: China Mechanical Criteria 20143402058). PCR reactions were performed in a real-time PCR thermocycler on the bench in BSL-2 and the results were read automatically. All materials that came into contact with clinical samples were immediately soaked in a 0.5 % chlorine-containing disinfectant for at least 10 min. Waste from the BSL-3 lab was carefully wrapped in a biohazard bag and sterilised twice, the first round of sterilisation was performed in an autoclave in BSL-3 laboratory, then transferred to another autoclave in the decontamination room via the “dirty” transfer window for a second round of sterilisation. Waste from the BSL-2 lab was sterilised directly in the autoclave in the decontamination room. All sterilised waste was incinerated.

## Safe and appropriate personnel protection equipment (PPE)

All personnel authorized to enter the laboratory must be well trained. Staff entering the yellow zone must wear at least disposable protective clothing, a disposable medical protective mask, a disposable hat, long-sleeved medical rubber gloves and overshoes. Staff entering the BSL-3 lab must wear a long-sleeved inner gown, a disposable hat, lab stockings, an N95 mask, inner-layer gloves, medical protective clothing, long-sleeved medical outer gloves and lab shoes in the 1^st^ changing room, and overshoes and long waterproof boots covers in the shower room, a positive-pressure breathing protection device and disposable waterproof clothing in the 2^nd^ changing room. Fit test must be done for all staffs that use the N95 mask. When leaving the BSL-3 lab, staff should remove the disposable waterproof clothing and replace the outer-layer gloves within the core area. The exterior layer of the protective clothing, the boot covers and the outer-layer gloves should be disinfected with 0.5 % chlorine-containing disinfectant for at least 10 min in the 2^nd^ changing room, and then the positive-pressure breathing protection device, medical protective clothing, boot covers and outer gloves were carefully removed. The N95 mask, disposable hat, overshoes and inner-layer gloves should be removed in the shower room. In the 1^st^ changing room, staff should remove the remaining inner PPE and change into their own clothes. PPE removal in the BSL-3 region and the 2^nd^ changing room should be carefully monitored by a working partner or by the staffs in the central control room through the emergency window (Fig. 1).

## Guarantee of biosafety and EBOV RNA-free environment

In 2015, between 11 March and 20 April, a total of 913 specimens were received in the SLE-CHN Biosafety Lab, including 350 blood specimens and 563 throat swabs. Among them, 44 were positive for EBOV, and the positive rates were 11.71 % in blood samples (41/350) and 0.53 % (3/563) in throat swab samples.

Based on the quality assurance requirement, contamination of the laboratory region with EBOV RNA should be evaluated by real-time RT-PCR every 2 months, so the first evaluation had been done at the end of the mission. Environmental specimens were collected with swabs, including (1) the surface of sample acceptance desk, (2) the temporary storage refrigerator, (3) the operation platform of biosafety cabinet in BSL-3 lab (before and after use), (4) the RNA extraction bench in the BSL-3 lab (before and after use), (5) the autoclave in the BSL-3 lab (inside and outside), (6) the centrifuge in the BSL-3 lab (inside and outside), (7) the transfer windows in the BSL-3 lab (“clean” and “dirty”), (8) the bench in the preparation room (before and after use), (9) the operation platform of biosafety cabinet in the BSL-2 lab (before and after use) and (10) the real-time PCR facility in BSL-2 (inside and outside). Each specimen was tested in duplicate. All samples were EBOV negative, indicating that the environment was EBOV RNA-free.

## Conclusion

Any improperly performed operations, from the acceptance of specimens to PCR preparation, will result in the possibility of EVD laboratory infection or environmental contamination with EBOV RNA, making test results unreliable. The absence of EBOV RNA in the laboratory environmental samples in this study reflects the importance of good laboratory practice for control of potential contamination with viral nucleic acids.

Therefore, appropriate PPE and good practices are essential for protecting medical and laboratory staff dealing with EVD. We believe that adequate preparation, comprehensive risk assessment and operation documents, appropriate PPE, effective monitoring and intensive training, together with well-designed and reasonable sectioning of the biosafety laboratory, are essential for guaranteeing biosafety.

## Abbreviations

BSL-3, biosafety level 3; EBOV, Ebola virus; EVD, Ebola virus disease; PPE, personal protection equipment; RT-PCR, reverse-transcription polymerase chain reaction; SLE-CHN Biosafety Lab, Sierra Leone-China Friendship Biological Safety Laboratory

## Additional file

Additional file 1: Multilingual abstracts in the six official working languages of the United Nations. (PDF 399 kb)
