# Supplementary material for: Good laboratory practices guarantee biosafety in the Sierra Leone-China friendship biosafety laboratory
Source: Infect Dis Poverty. 2016 Jun 23;5:62. doi: 10.1186/s40249-016-0154-5 (PMC4918109; doi:10.1186/s40249-016-0154-5)
Supplement: Additional file 1: — Multilingual abstracts in the six official working languages of the United Nations. (PDF 399 kb) [file 40249_2016_154_MOESM1_ESM.pdf]

Translation of the abstract into the five official working languages of the United Nations

الممارسات المخبرية الجيدة تضمن السلامة البيولوجية في مختبرالتعاون للسلامة البيولوجية بين سيراليون و الصين

كين وانغ- وي من زو- يونغ زانغ هانغ- هوانيو وانغ- هاي جان دو- كاي ني- جينغ دونغ سونغ- كانغ اكيو- وين وين لي- جيان كيانغ غوا- هي جيانغ وي- كون كاي- يانغ هاي وانغ- جيانغ ويو- جيرارد كامارا- ايدريسا كامارا- كيانغ وي- مي فانغ ليانغ- غوي زن ويو- اكسيو بينغ دونغ

ملخص

نبذة عن الدراسة: يعتبرتفشي فيروس ايبولا في غرب افريقيا بين عامي 2014- 2015 من أكبر الأوبئة منذ ان تم التعرف على هذا الفيروس في عام 1976. ومن أكثر البلدان تأثرا بهذا الفايروس هي سيراليون وغينيا ولايبيريا.

النتائج: أسست الحكومة الصينية مختبر التعاون بين سيراليون والصين للسلامة البيولوجية وهو مختبر من الدرجة الثالثة للسلامة البويلوجية الثابتة ومقره العاصمة سيراليون. ولا يزال العمل مستمر في هذا المختبر منذ تفشي هذا الفيروس في الحادي عشر من شهر اذار من عام 2015. وقد تم انشاء ادارة ووثائق البرنامج كاملة لمختبر التعاون بين سيراليون والصين للسلامة البيولوجية. وتم تقسيم هذا المختبر الى أربعة مناطق وهي كالتالي: المنطقة الخضراء والصفراء والبنية والحمراء. وقد تم هذا التقسيم حسب تقييم الخطورة. ويتم استخدام أنواع مختلفة من معدات الحماية الامنة والمناسبة حسب لون المنطقة في المختبر. يتبع هذا التصنيف معايير منظمة الصحة العالمية للسلامة الأحيائية في المختبرات من الدرجة الثالثة.

الخلاصة: يعتبر الاستعداد الجيد ووثائق العملية وتقييم الخطورة الشامل واستخدام معدات الحماية المناسبة والمراقبة الفعالة والتدريب المكثف الى جانب التقسيم المصمم بشكل جيد ومعقول من النقاط الضرورية لضمان السلامة الأحيائية في المختبرات.

Translated from English version into Arabic by Randa82, through

TRANSLATORS
WITHOUT BORDERS

健全的实验室规范是中塞友好生物安全实验室生物安全的保障

王芹，周为民，张勇，王环宇，杜海军，聂凯，宋敬东，肖康，雷雯雯，郭建强，隗合江，蔡琨，王衍海，吴江，Gerard Kamara, Idrissa Kamara，魏强，梁米芳，武桂珍，董小平

摘要

引言：自 1976 年埃博拉病毒（EBOV）被发现以来，2014~2015 年埃博拉病毒病在西非出现了有史以来最大的一次暴发疫情，西非三国（塞拉利昂、几内亚和利比里亚）遭到了重创。

结果：建立在塞拉利昂首都弗里敦的中塞友好生物安全实验室是中国政府援建的一座固定生物安全三级实验室，该实验室于 2015 年 3 月 11 日启动 EBOV 检测工作。中塞友好生物安全实验室创建了完备的体系文件。整个实验室根据风险评估分成四个区：绿色区、黄色区、棕色区和红色区。在实验室不同的区域，工作人员使用不同类型的个人防护装备，完全符合世界卫生组织关于生物安全三级实验室的标准。

结论：充分的准备工作、全面细致的风险评估和操作文件、合适的个人防护装备、有效的监控及强化的训练，配以精心设计和合理的实验室布局，是保证中塞友好生物安全实验室生物安全的关键。

Translated from English version into Chinese by Yong Zhang

De bonnes pratiques en laboratoire garantissent la sécurité biologique dans le laboratoire de l’amitié sino-

sierra- léonaise en matière de bio sécurité

Qin Wang, Wei-Min Zhou, Yong Zhang, Huan-Yu Wang, Hai-Jun Du, Kai Nie, Jing-Dong Song, Kang Xiao, Wen-Wen Lei, Jian-Qiang Guo, He-Jiang Wei, Kun Cai, Yan-Hai Wang, Jiang Wu, Gerard Kamara, Idrissa Kamara, Qiang Wei, Mi-Fang Liang, Gui-Zhen Wu, Xiao-Ping Dong

Résumé

Historique : L'épidémie de la maladie du virus Ebola (EVD) en Afrique de l'Ouest entre 2014 à 2015 fut la plus grande épidémie d'Ebola depuis la découverte du virus Ebola (EBOV) en 1976. La Sierra-Léone, la Guinée et le Libéria furent les pays les plus touchés par la maladie.

Observations: Le laboratoire de l'amitié sino-sierra-léonaise en matière de sécurité biologique (SLE-CHN laboratoire), un laboratoire de sécurité biologique de niveau sanitaire 3 a été implanté dans la capitale de Sierra-Leone par le gouvernement chinois ; le laboratoire s'est montré actif dans la découverte du virus Ebola (EBOV) depuis le 11 mars 2015. Une gestion complète et les documents du programme ont été élaborés pour le laboratoire de biosécurité SLE-CHN qui a été réparti en quatre zones (les zones verte, jaune, marron et rouge) en fonction de l'évaluation des risques. Du matériel varié en matière de sécurité ainsi que des équipements appropriés pour le personnel sont utilisés dans les différentes zones du laboratoire et celui-ci répond totalement aux critères de niveau sanitaire 3 d'un laboratoire en matière de biosécurité de l'Organisation Mondiale de la Santé (OMS).

Conclusion : Une bonne préparation, une évaluation complète des risques et des documents opérationnels, un matériel de protection individuel approprié (PPE), un suivi efficace et une formation intensive, liés à une répartition appropriée et bien conçue du laboratoire, sont essentiels pour garantir la sécurité biologique

Translated from English version into French by Veromarie, through

Надлежащая лабораторная практика обеспечивает соблюдение норм биобезопасности в Лаборатории Биологической Безопасности имени Дружбы Народов Сьерра-Леоне и Китая.

Цинь Ван, Вэй-Мин Чжоу, Юн Чжан, Хуань-Ю Ван, Хай-Жунь Ду, Кай Не, Цзин-Дун Сун, Кан Сяо, Вэнь-Вэнь Лэй, Цзянь-Цян Го, Он-Цзян Вэй, Кун Цай, Ян-Хай Ван, Цзян Ву, Джерард Камара, Идрисса Камара, Цян Вэй, Ми-Фан Лян, Гуй-Жень Ву, Сяо-Бин Дун

Краткий обзор

Общие сведения: Вспышка болезни, вызванной вирусом Эбола (БВВЭ), в Западной Африке в 2014-2015 годах была крупнейшей эпидемией БВВЭ с момента идентификации вируса Эбола (EBOV) в 1976 году, от которой наиболее сильно пострадало население стран Сьерра-Леоне, Гвинеи и Либерии.

Выводы: Лаборатория Биологической Безопасности им. Дружбы Народов Сьерра-Леоне и Китая (Лаборатория Биобезопасности SLE–CHN), находящаяся в столице Сьерра-Леоне и имеющая штатный уровень биологической безопасности 3, была создана китайским правительством, и с 11 марта 2015 активно занимается выявлением EBOV. Для лаборатории Биобезопасности SLE–CHN был создан полный пакет

контрольно-организационной и программной документации, и лаборатория, согласно оценке степени рисков, была разделена на четыре зоны (зеленую, желтую, коричневую и красную). В разных зонах лаборатории используются различные средства индивидуальной защиты (СИЗ), которые в полной мере отвечают лабораторным стандартам Всемирной Организации Здравоохранения для уровня биологической безопасности 3.

Заключение: Для гарантии соблюдения норм биологической безопасности необходима тщательная подготовка, наличие полного комплекта документов по оценке степени рисков и норм эксплуатации, эффективные СИЗ, действенный оперативный контроль и всестороннее обучение в сочетании с продуманным и обоснованным разграничением зон лаборатории.

Translated from English version into Russian by Tatiana Petrosyan, through

Las prácticas de laboratorio garantizan la bioseguridad en el Laboratorio de Bioseguridad de la Amistad Sierra Leona - China

Qin Wang, Wei-Min Zhou, Yong Zhang, Huan-Yu Wang, Hai-Jun Du, Kai Nie, Jing-Dong Song, Kang Xiao, Wen-Wen Lei, Jian-Qiang Guo, He-Jiang Wei, Kun Cai, Yan-Hai Wang, Jiang Wu, Gerard Kamara, Idrissa Kamara, Qiang Wei, Mi-Fang Liang, Gui-Zhen Wu, Xiao-Ping Dong

Resumen

Antecedentes: El brote de la enfermedad por el virus del ébola (EVE) que se produjo en África Occidental entre los años 2014 y 2015 fue la mayor epidemia de EVE desde que se identificara el virus del ébola en 1976, y los países más gravemente afectados fueron Sierra Leone, Guinea y Liberia.

Observaciones: El Laboratorio de Bioseguridad de la Amistad Sierra Leona - China, un laboratorio de bioseguridad de nivel 3 situado en la capital de Sierra Leona, fue establecido por el gobierno chino y viene trabajando activamente en la detección del virus del ébola desde el 11 de marzo de 2015. Se elaboraron una serie completa de documentos de gestión y programa para el Laboratorio de Bioseguridad Sierra Leona - China, y se dividió este en cuatro zonas (verde, amarilla, marrón y roja) según el nivel de riesgo de cada una. En las diferentes zonas del laboratorio se utilizan distintos tipos de equipos de seguridad y protección para el personal, y se cumple cabalmente con los estándares establecidos por la Organización Mundial de la Salud para los laboratorios de bioseguridad de nivel 3.

Conclusión: La correcta preparación, los documentos de evaluación integral de riesgos y de funcionamiento, la protección apropiada para el personal, el monitoreo eficaz y la capacitación intensiva, junto con una división bien diseñada y racional del laboratorio son esenciales para garantizar la bioseguridad.

Translated from English version into Spanish by Mónica Algazi, through

TRANSLATORS
WITHOUT BORDERS
